# Diurnal changes in the murine small intestine are disrupted by obesogenic Western Diet feeding and microbial dysbiosis

**DOI:** 10.1038/s41598-021-98986-7

**Published:** 2021-10-18

**Authors:** Sarah E. Martchenko, David Prescott, Alexandre Martchenko, Maegan E. Sweeney, Dana J. Philpott, Patricia L. Brubaker

**Affiliations:** 1 Departments of Physiology, University of Toronto, Rm 3366 Medical Sciences Building, 1 King’s College Circle, Toronto, ON M5S 1A8 Canada; 2 Department of Immunology, University of Toronto, Toronto, ON Canada; 3 Department of Laboratory Medicine and Pathobiology, University of Toronto, Toronto, ON Canada; 4 Department of Medicine, University of Toronto, Toronto, ON Canada

**Keywords:** Physiology, Gastroenterology

## Abstract

Intestinal functions demonstrate circadian rhythms thought to be entrained, in part, by an organisms’ intrinsic feeding and fasting periods as well as by the intestinal microbiome. Circadian disruption as a result of ill-timed nutrient exposure and obesogenic feeding poses an increased risk to disease. As such, the aim of this study was to assess the relationships between dietary timing, composition, and the microbiome with regard to rhythmic small intestinal structure and mucosal immunity. Rodent chow (RC)-mice exhibited time-dependent increases in small intestinal weight, villus height, and crypt depth as well as an increased proportion of CD8αα^+^ cells and concomitant decrease in CD8αβ+ cells at the onset of the feeding period (*p* < 0.05–0.001). Western diet (WD)-animals displayed disrupted time-dependent patterns in intestinal structure and lymphocyte populations (*p* < 0.05–0.01). Antibiotic-induced microbial depletion abrogated the time- and diet-dependent patterns in both RC- and WD-mice (*p* < 0.05–0.001). However, although germ-free-mice displayed altered rhythms, fecal microbial transfer from RC-mice was generally unsuccessful in restoring structural and immune changes in these animals. This study shows that adaptive changes in the small intestine at the onset of the feeding and fasting periods are disrupted by WD-feeding, and that these changes are dependent, in part, on the intestinal microbiome.

## Introduction

While circadian rhythms have developed over evolutionary time as an adaptive response to external light–dark cycles, peripheral tissues, including the gastrointestinal (GI) tract, exhibit diurnal rhythms in the absence of direct exposure to these cycles^1,2^. Hence, although the GI tract does receive input from the master clock expressed within the suprachiasmatic nuclei, the gut intestinal epithelial cells (IECs) also demonstrate self-sustained rhythmic expression of the circadian clock genes that are entrained by extrinsic cues originating from both ingested nutrients and the microbiome^3–11^. These rhythms are established by a transcriptional-translational feedback loop between the positive (i.e. *Arntl* (aka *Bmal1*)/*Clock*) and negative (i.e. *Per1-3*/*Cry1-2*) arms of the core clock^12^, and have been estimated to affect the expression of 43% of the genes in the mammalian genome^13^.

Over several decades, circadian misalignment due to the disruption of clock gene expression has been linked to impaired human health. Hence, ill-timed exposure to light and/or nutrients leads to an increased risk for metabolic disease, as documented in individuals who conduct shift work and in normal rodents exposed to constant light or obesogenic diets^12,14–16^. In parallel, clock gene mutant rodent models also demonstrate impaired metabolic homeostasis^17–21^. However, both GI dysfunction and microbial dysbiosis have been also reported for humans undergoing shift work and rodents subjected to food-, light- or genetically-induced circadian disruption^5,7,8,22–25^. Consistent with these findings, IEC proliferation also follows a 24-h rhythm, which is abolished in *Clock* mutant and *Arntl* knockout mice^26,27^. Furthermore, important digestive and absorptive functions of the intestine, as well as barrier integrity, all follow a circadian rhythm as a result of clock-controlled expression of key enzymes, transporters, and tight junction proteins^11^.

While the majority of the small intestinal epithelium is comprised of nutrient-absorbing enterocytes, intraepithelial lymphocytes (IELs) dispersed between the IECs function to establish mucosal immune responses against invading pathogens and to maintain epithelial integrity^9,28^. The other major intestinal lymphocyte population resides in the lamina propria, deemed lamina propria lymphocytes (LPLs). Like IELs, LPLs are critical for defense against intestinal pathogens, but they also serve a role in establishing the IEL cell population through specific changes in homing markers^28^. Diurnal patterns in the immune system have been demonstrated in several peripheral tissues, including the intestine, whereby the number of immune cells is reduced during the fasting period through the induction of apoptosis^29^. The circadian regulation of immune cell populations in the intestine is believed to result, at least in part, from immune responsiveness to dietary antigen exposure following food intake^9,29^. However, the factors that regulate the daily patterns in IELs and LPLs remain of great interest, as the SI must maintain dietary antigenic tolerance while simultaneously providing immune protection during the rhythmic cycles of feeding and fasting.

Recent findings have demonstrated that both the IECs and the intestinal immune system are profoundly influenced by the microbiome^4,5,8,9^. Germ-free (GF) mice display reduced IEC proliferation in association with a thinning of the intestinal mucosa^30^. Intestinal microbes have been further demonstrated as essential for intestinal repair, as mice deficient for innate immune toll-like receptors exhibit reduced IEC proliferation, survival, and barrier function^31^. Moreover, the intestinal microbiome has been well documented as a fundamental regulator of intestinal lymphocyte localization and function^32^. Furthermore, recent evidence supports the notion that disruption of the microbiome through dietary changes or antibiotic use leads to a perturbation in overall SI homeostasis, including disrupted gene expression, barrier integrity, and cytokine production^5,6,9^.

While there appears to be an essential interaction between the circadian rhythms expressed by the intestinal epithelium, the intestinal lymphocytes, and the intestinal microbiome, the exact relationship between these key determinants of GI function remains unclear. The goal of the present study was, therefore, to interrogate the effects of both diet and microbial dysbiosis on circadian intestinal homeostasis. Thus, at the onset of the active/feeding and rest/fasting periods, regular chow (RC)- and high-fat/high-sucrose Western diet (WD)-fed mice, as well as RC- and WD-animals with antibiotic-induced microbial disruption (AIMD) and GF-mice with and without fecal microbial transfer (FMT) from RC-mice were used to characterize the intimate relationship between an intact microbiome, intestinal structure and mucosal immunity. The findings indicate that time- and diet-dependent changes in SI gravimetric and morphometric parameters as well as in intestinal lymphocyte populations are, at least in part, dependent on an intact intestinal microbiome.

## Results

### The intestinal microbiome regulates time- and diet-dependent structural changes in the small intestine

**Figure 1:**
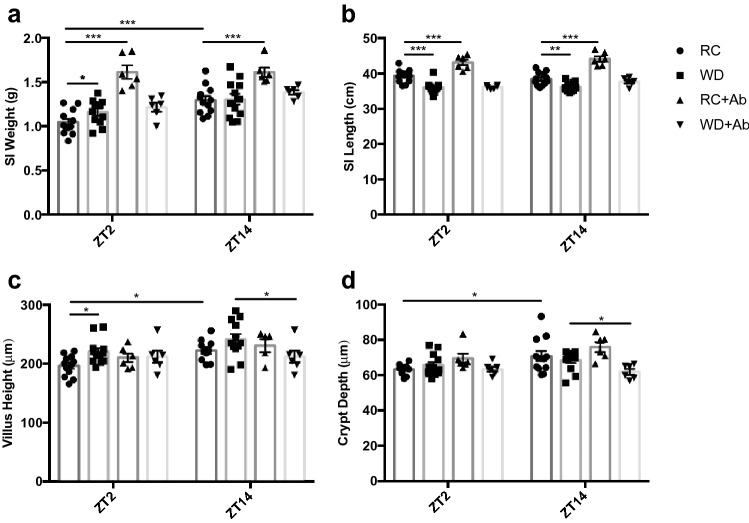
WD-feeding and microbial disruption alter time-dependent patterns in SI gravimetric and morphometric parameters. (**a**) SI weight, (**b**) SI length, (**c**) ileal villus height, and (**d**) ileal crypt depth in RC- and WD-fed animals with and without AIMD measured at ZT2 and ZT14. n = 3–6 male and 3–6 female mice. *p < 0.05 **p < 0.01 ***p < 0.001.

To characterize time-dependent changes in the SI of normal mice, RC-fed, male and female mice were examined at the onset of the established rodent fasting and feeding periods, Zeitgeber times 2 and 14 (ZT; hours after lights-on), respectively. SI wet weight increased by 19% at ZT14 as compared ZT2, with no differences by time observed in SI length (Fig. 1a and b). To account for the time-of-day differences in SI weight independent of length, ileal villus height and crypt depth were also determined. Villus height as well as crypt depth were significantly increased at ZT14 as compared to ZT2 by 12% and 11%, respectively (Fig. 1c and d; Supplementary Figure 1). Together, the increases in intestinal weight, villus height, and crypt depth at ZT14 suggest anticipatory changes in the SI mucosa in preparation for the onset of the animals’ feeding period.

To establish the effects of dietary alterations on the gravimetric and morphometric changes in the SI with time-of-day, mice were fed a WD for 16 weeks. Unexpectedly, the increase in SI weight between ZT2 and ZT14 that was observed in RC-animals was lost in the WD-fed mice, as a result of increased weight at ZT2 (Fig. 1a). Furthermore, an overall decrease in SI length was observed in these animals at both time points (Fig. 1b). In keeping with loss of the rhythm in SI weight, villus height in WD-animals was also increased at ZT2, with no differences observed in crypt depth (Fig. 1c and d; Supplementary Figure 1). These findings suggest the existence of diet-driven temporal effects on SI structure.

As one potential regulator of the time- and diet-dependent changes in the SI is the intestinal microbiome, depletion of the microbiome was accomplished by administration of antibiotics (AIMD). As previously reported at the phyla level in these animals^33^, microbial dysbiosis in the RC AIMD-animals was characterized by a massive increase in the relative abundance of colonic *Proteobacteria* as compared to RC mice. Parallel analysis at the family level confirmed microbial disruption as RC AIMD mice were predominantly colonized by *Burkholderiaceae* (Supplementary Figure 2). RC-fed AIMD-mice were also found to have increased SI weight and length at both ZT2 and 14, along with abrogation of their normal diurnal rhythm in SI weight (Fig. 1a and b). Interestingly, this was accompanied by a loss in the normal time-dependent difference in villus height but, again, with no changes in crypt depth (Fig. 1c and d; Supplementary Figure 1). In contrast, antibiotic-treated WD-fed mice, the colons of which were colonized almost exclusively by the *Streptococcaceae* family (Supplementary Figure 2), a member of the *Firmicutes* phylum^33^, did not demonstrate the increases in SI weight and length that were observed in RC AIMD-animals (Fig. 1a and b) and, indeed, demonstrated decreased villus height and crypt depth at ZT14 as compared to WD-fed mice (Fig. 1c and d; Supplementary Figure 2). Together, these findings suggest a role for the microbiome in both the time- and diet-dependent changes in SI structure.

**Figure 2:**
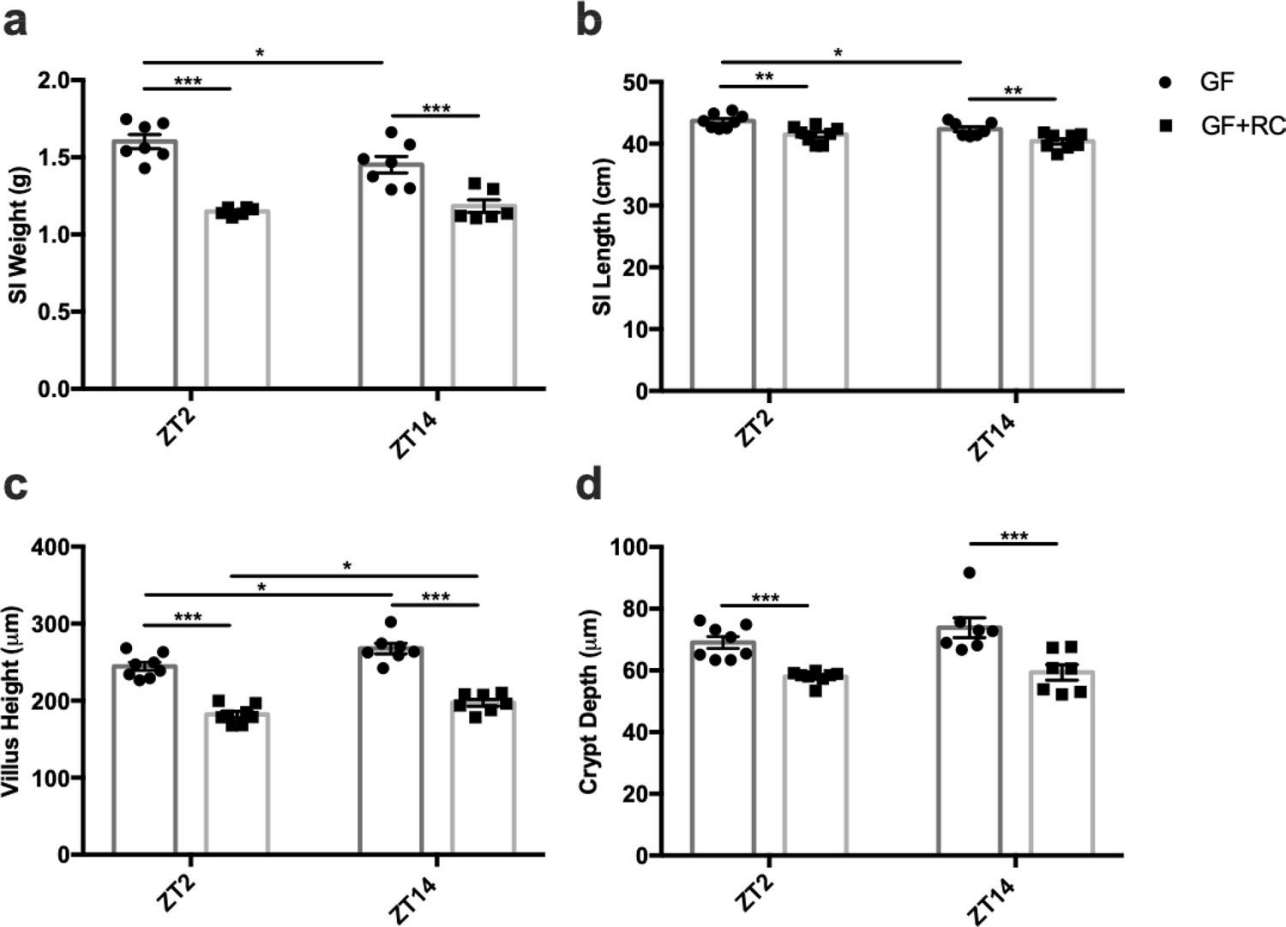
FMT reduces SI gravimetric and morphometric parameters independent of time. (**a**) SI weight, (**b**) SI length, (**c**) ileal villus height, and (**d**) ileal crypt depth in GF and GF-mice following FMT with RC-microbiome (GF + RC) measured at ZT2 and ZT14. n = 3–4 male and 3–4 female mice. *p < 0.05 **p < 0.01 ***p < 0.001.

To establish the essentiality of the microbiome in the structural changes in the SI with time, GF-mice were examined without (control) and with transplantation of feces from conventional RC-mice (GF + FMT). Successful recolonization was achieved as evidenced by the presence of major phyla^33^ and families (Supplementary Figure 3) that were observed in normal mice. However, it is important to note that the relative abundance of select families differed between the RC and GF + FMT mice (Supplementary Figure 2 and 3). GF-mice displayed SI weights and lengths that were comparable to those observed in RC-AIMD animals (Fig. 2a and b vs Fig. 1a and b) and, as found in the antibiotic-treated mice, did not demonstrate the normal time-dependent changes in SI weight. Interestingly, FMT into the GF-mice reduced both SI weight and length to levels that were comparable to those observed in conventional RC-animals; however, SI weight did not regain the time-dependent difference between ZT2 and ZT14. Consistent with the findings made for SI weight, GF-mice demonstrated villi and crypts that appeared slightly longer than normal (Fig. 2c and d vs. Figure 1c and d; Supplementary Figure 4), although the normal time-dependent pattern in villus height was maintained. Furthermore, both villus height and crypt depth were significantly reduced in GF + FMT mice, to levels that were comparable to those observed in the RC-animals, with maintenance of the temporal pattern in villus height. When taken with findings in the AIMD animals, these results demonstrate a key role for the microbiome in the establishment of temporal patterns in SI structure.

### The intestinal microbiome regulates time- and diet-dependent changes in small intestinal gene expression

**Figure 3:**
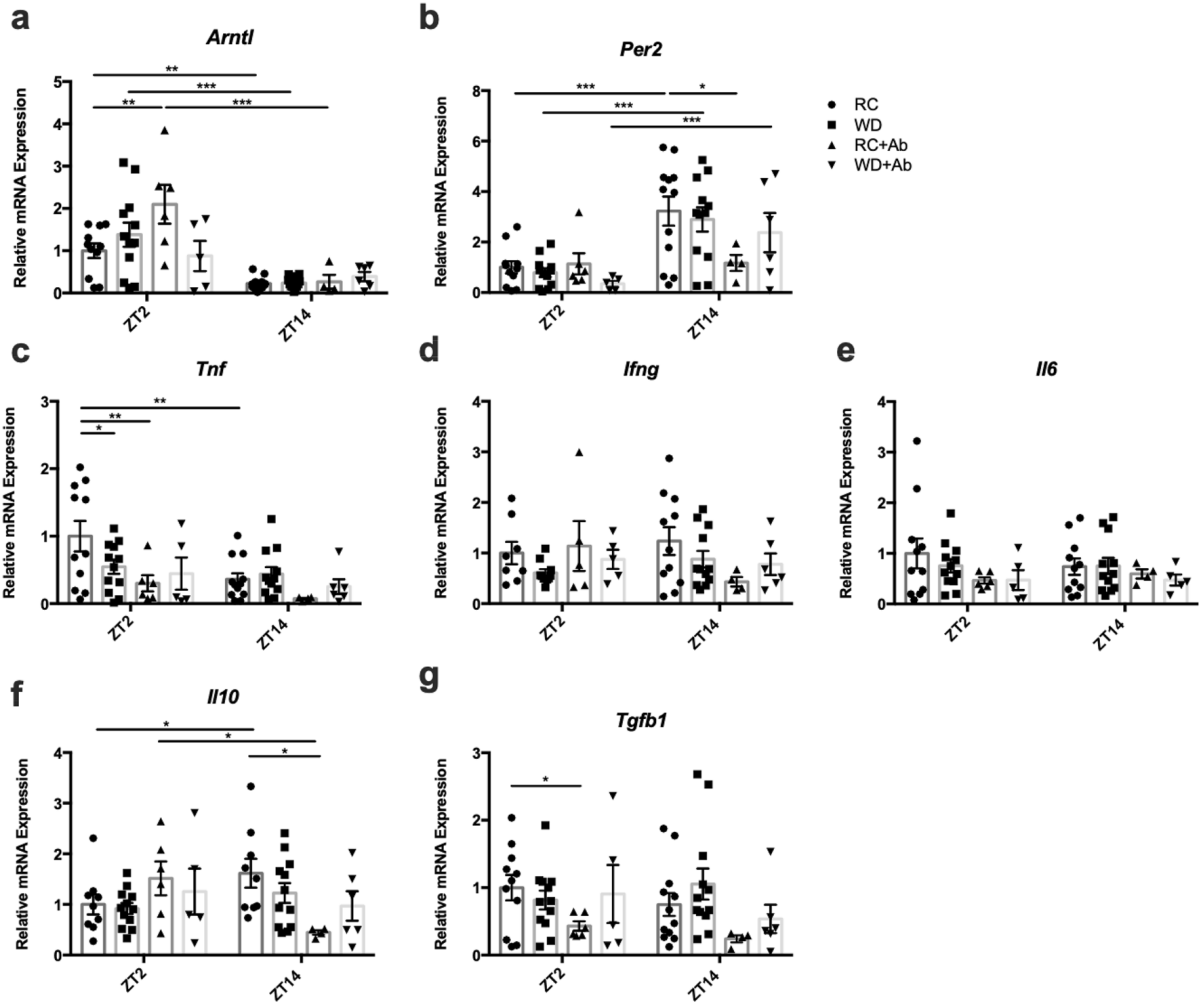
Intestinal mucosal gene expression is altered by WD-feeding and microbial disruption. (**a**) *Arntl*, (**b**) *Per2*, (**c**) *Tnf*, (**d**) *Ifng*, (**e**) *Il6*, (**f**) *Il10*, and (**g**) *Tgfb* mRNA expression measured from SI mucosa isolated at ZT2 and ZT14 from RC- and WD-fed animals with and without AIMD. n = 2–6 male and 2–6 female mice. *p < 0.05 **p < 0.01 ***p < 0.001.

Given that both diet and the intestinal microbiome have been shown to alter the intestinal epithelial circadian clock and immune homeostasis^4–9,33^, expression of the core clock genes, *Arntl* and *Per2* as well as several markers of intestinal inflammation (*Tnf, Ifng, Il6, Il10,Tgfb1*) were examined in the SI mucosa. In line with previous evidence^33,34^, *Arntl* and *Per2* exhibited anti-phasic expression in normal mouse SI, with greater expression of *Arntl* at ZT2 as compared to ZT14, and of *Per2* at ZT14 versus ZT2 (Fig. 3a and b). Although previous reports indicate that obesogenic diets disrupt metabolic tissue clock gene expression^35,36^, including the intestinal enteroendocrine L cell^33^, WD-feeding did not significantly alter the levels of, or patterns in mucosal clock gene expression. However, microbial dysbiosis disrupted SI clock gene expression in both RC- and WD-fed mice, in a diet-dependent manner (Fig. 3a and b). Hence, RC-AIMD animals demonstrated increased *Arntl* expression at ZT2 and a complete loss of rhythmic *Per2* expression, whereas WD-AIMD mice lost rhythmic *Arntl* expression (Fig. 2a and b).

Analysis of select immune cytokines also revealed time-dependent expression of genes that was differentially affected by diet and AIMD. RC SI mucosal *Tnf* gene expression demonstrated decreased expression at ZT14 as compared to ZT2, a pattern that was lost with both WD-feeding and AIMD (Fig. 3c). Expression of other pro-inflammatory cytokines, *Ifng* and *Il6*, did not display time-dependent differences in RC-mice and was not significantly altered by feeding or AIMD (Fig. 3d and e). In contrast to *Tnf*, the anti-inflammatory cytokine, *Il10*, had elevated expression at ZT14 compared to ZT2 in RC-fed mice only, which was reversed by AIMD (Fig. 3f). Furthermore, *Tgfb1* expression was largely unaffected by diet, but was significantly reduced in RC-AIMD mice at the ZT2 time point, with a similar trend at ZT14 (Fig. 3g).

**Figure 4:**
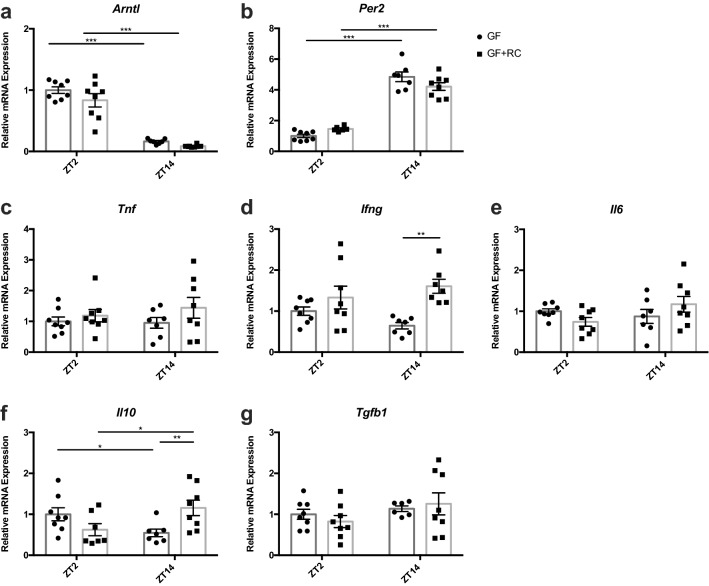
Intestinal﻿ mucosal gene expression is partially restored following FMT. (**a**) *Arntl*, (**b**) *Per2*, (**c**) *Tnf*, (**d**) *Ifng*, (**e**) *Il6*, (**f**) *Il10*, and (**g**) *Tgfb* mRNA expression measured from SI mucosa isolated at ZT2 and ZT14 from GF and GF-mice following FMT with RC-microbiome (GF + RC) measured at ZT2 and ZT14. n = 3–4 male and 3–4 female mice. *p < 0.05 **p < 0.01 ***p < 0.001.

Interestingly, in the complete absence of the microbiome, GF-animals maintained rhythmic anti-phasic *Arntl* and *Per2* expression, which was not altered following FMT (Fig. 4a and b). In contrast, GF-mice did not demonstrate the normal patterns in *Tnf* and *Il10* that were observed in RC-mice (Fig. 4c–g), although *Il10* did regain rhythmic expression in GF + FMT mice, with increased levels at ZT14 (Fig. 4f). The minimal changes in gene expression seen following FMT in GF-mice may speak to the need for a microbial-immune interaction from birth^32^.

### Differential effects of obesogenic feeding and microbial disruption on the diurnal patterns in intestinal lymphocytes

**Figure 5:**
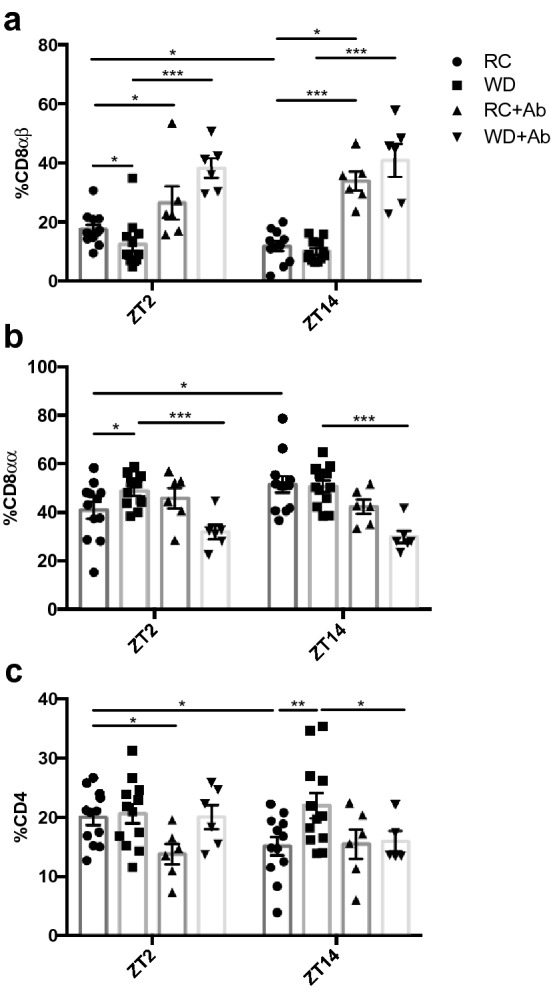
Time, diet, and microbial disruption alter proportions of SI IEL cell populations. Proportions of (**a**) CD8αβ^+^, (**b**) CD8αα^+^, and (**c**) CD4^+^ on TCRβ^+^ cells acquired from the SI epithelium at ZT2 and ZT14 from RC- and WD-fed animals with and without AIMD. n = 3–6 male and 3–6 female mice. *p < 0.05 **p < 0.01 ***p < 0.001.

To characterize time-, diet- and microbiome interactions within the intestinal immune cell population, the IEL and LPL compartments were characterized in RC- and WD-mice, with and without antibiotic treatment. Examination of the IEL profile revealed both time- and diet-dependent changes, which appeared to be largely dependent on the existence of an intact intestinal microbiome. Thus, the proportion of CD8αβ^+^ IELs was elevated at ZT2 as compared to ZT14 in RC-mice, whereas WD-mice lacked a rhythm, having significantly reduced CD8αβ^+^ at ZT2 (Fig. 5a). AIMD significantly increased the proportion of CD8αβ^+^ IELs in both RC- and WD-animals at both ZT2 and 14, resulting in a loss of the normal rhythm. Furthermore, the highest proportions of CD8αβ^+^ IELs were observed in the WD-AIMD animals. In contrast, time-dependent differences in CD8αα^+^ IELs in RC-mice were opposite to those of the CD8αβ^+^ IELs, showing increased proportions at ZT14 (Fig. 5b). WD-feeding resulted in loss of rhythm in CD8αα^+^ IELs as a result of increased proportions observed at ZT2 (Fig. 5b). In addition, both RC- and WD-AIMD animals lost the rhythm in CD8αα^+^ IELs in association with a reduction in this cell population in WD-AIMD mice at both time points (Fig. 5b). As found for the CD8αβ^+^ IELs, the proportion of CD4^+^ cells was also decreased at ZT14 as compared to ZT2 in RC-mice, whereas WD-fed animals lost this rhythm in combination with increased CD4^+^ cells at ZT14 (Fig. 5c). AIMD in RC-mice also demonstrated reduced proportions of CD4^+^ cells at ZT2 while, in WD-fed AIMD-animals, this cell population was reduced at ZT14 (Fig. 5c).

**Figure 6:**
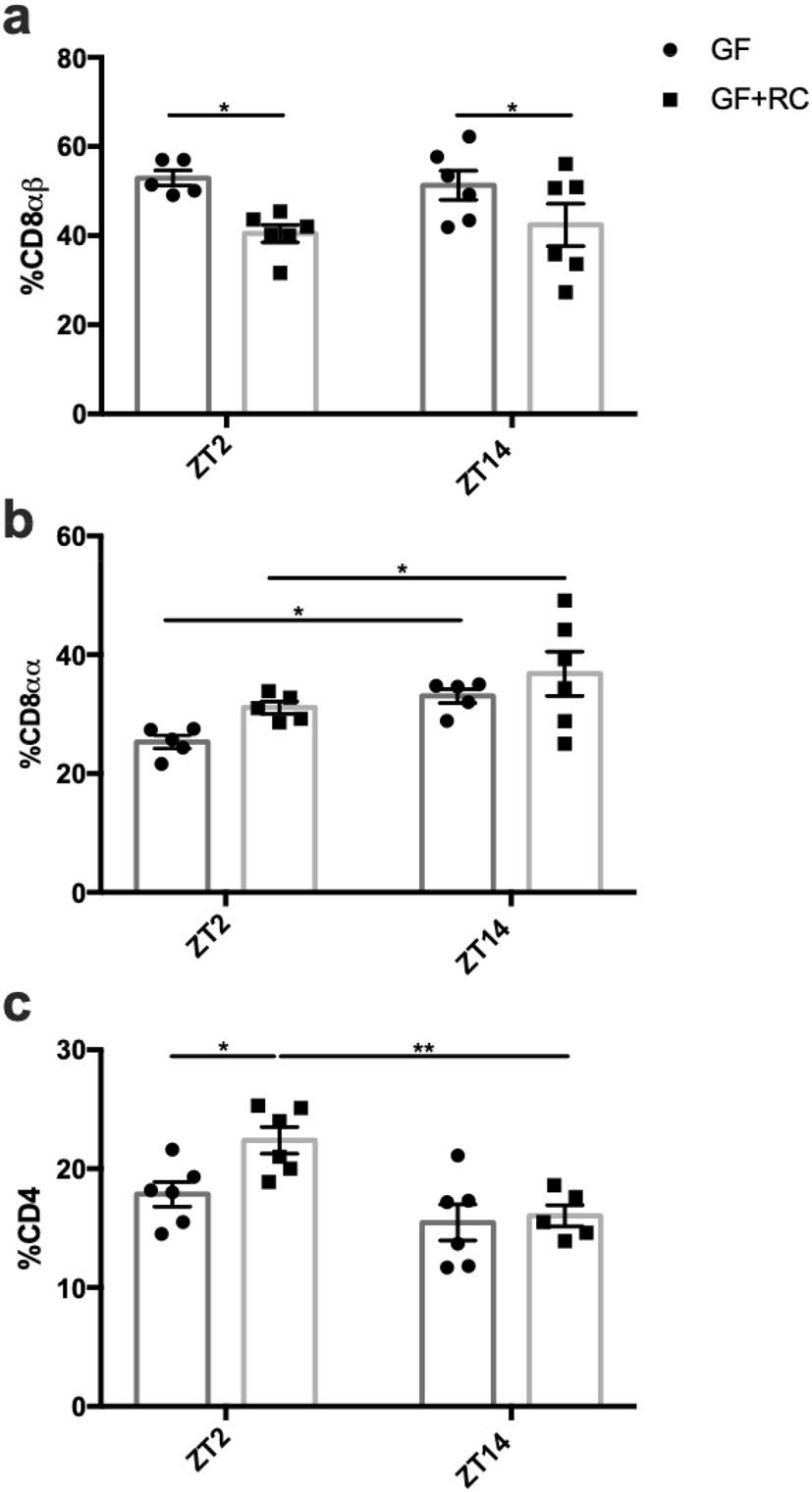
Time-dependent changes in SI IEL cell populations are partially restored following FMT in GF-mice. Proportions of (**a**) CD8αβ^+^, (**b**) CD8αα^+^, and (**c**) CD4^+^ on TCRβ^+^ cells acquired from the SI epithelium at ZT2 and ZT14 from GF and GF-mice following FMT with RC-microbiome. n = 2–3 male and 2–3 female mice. *p < 0.05 **p < 0.01.

Analysis of CD8αβ^+^ IELs in GF-mice revealed no time-dependent changes, although the overall proportions of these cells were greater than observed in either normal RC-mice or in RC-AIMD animals (Fig. 6a vs. Fig. 5a). Although, FMT failed to re-establish the rhythm in this cell population, the presence of a microbiome did reduce the overall proportion of these IELs at both time points (Fig. 6a). Interestingly, the time-dependent changes in the proportion of CD8αα^+^ IELs were maintained in both GF-mice and in GF-mice with FMT (Fig. 6b). However, the CD4^+^ IEL population lacked a significant rhythm in GF-mice, and this was restored following FMT (Fig. 6c).

**Figure 7:**
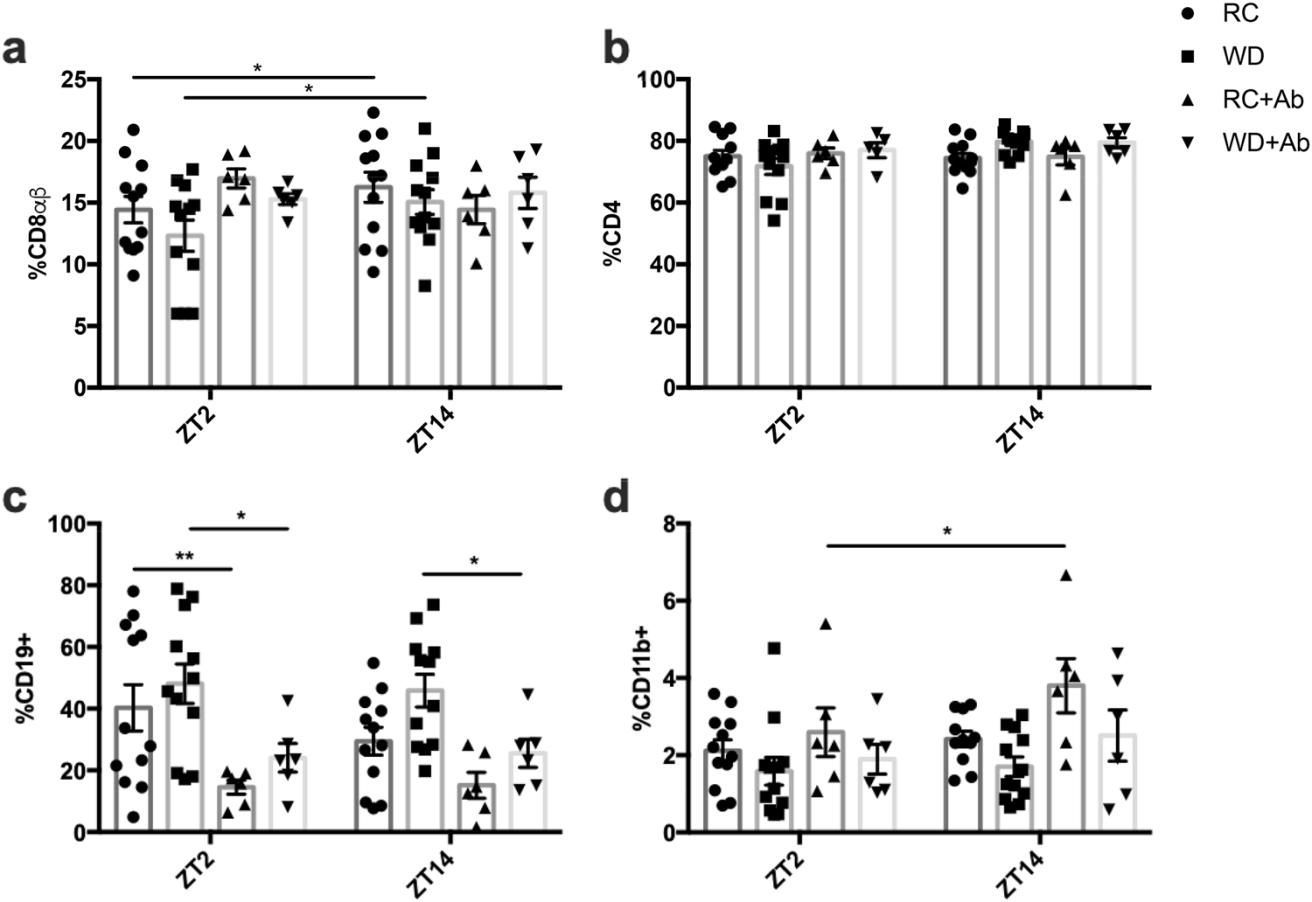
Time-dependent changes are lost in LPL %CD8αβ^+^ cells following microbial disruption. Proportions of (**a**) CD8αβ^+^and (**b**) CD4^+^ on TCRβ^+^ cells, as well as (**c**) CD19 + B-cells and (**d**) CD11b + myeloid cells acquired from the SI lamina propria at ZT2 and ZT14 from RC- and WD-fed animals with and without AIMD. n = 3–6 male and 3–6 female mice. * *p* < 0.05.

**Figure 8:**
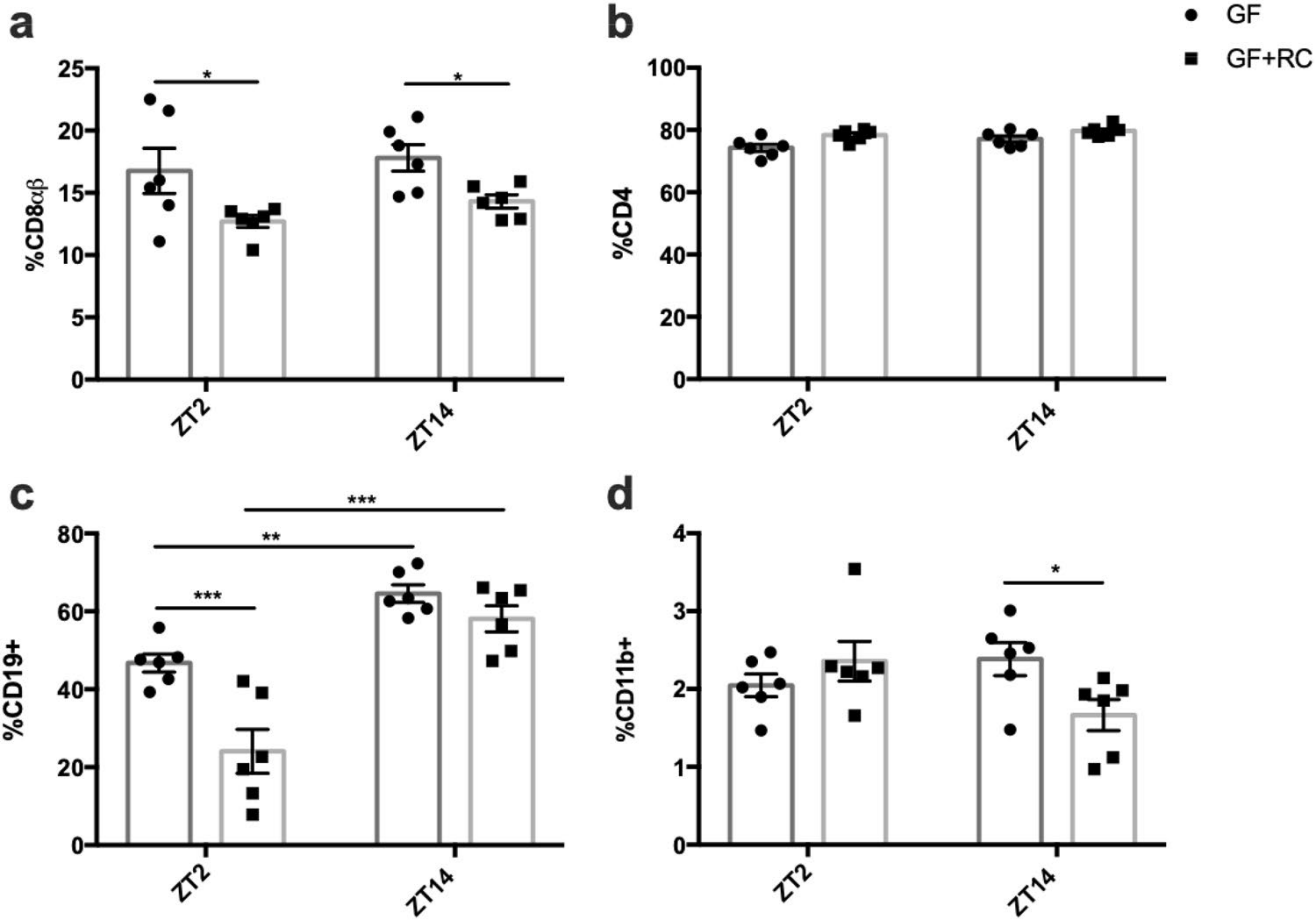
FMT in GF-mice alters proportion of CD8αβ^+^ but not CD4^+^ SI LPL cell populations. Proportions of (**a**) CD8αβ^+^and (**b**) CD4^+^ on TCRβ^+^ cells, as well as (**c**) CD19 + B-cells and (**d**) CD11b + myeloid cells acquired from the SI lamina propria at ZT2 and ZT14 from GF and GF-mice following FMT with RC-microbiome. n = 2–3 male and 2–3 female mice. *p < 0.05.

In contrast to the patterns observed in the IELs, the proportion of CD8αβ^+^ cells in the LPL compartment revealed opposing time-dependent changes in RC-mice, with an increase at ZT14 that was maintained upon WD-feeding (Fig. 7a). Interestingly, AIMD in both RC- and WD-fed mice resulted in loss of this rhythm (Fig. 7a). The CD4^+^ LPLs remained constant irrespective of time, diet, and microbial disruption (Fig. 7b). However, while analysis of the LPL B-cell (CD19 +) population revealed no time- or diet-dependent changes, consistent with previous reports^37^, antibiotic administration decreased the cell proportion under both diet conditions (Fig. 7c). Furthermore, no time-, diet-, or treatment-dependent differences were observed in the proportion of myeloid cells (CD11b +) within the LPL (Fig. 7d). Germ-free animals did not display time-dependent differences in CD8αβ^+^ IELs (Fig. 8a). However, as also observed for the proportion of CD8αβ^+^ IELs, FMT in GF-animals failed to re-establish the normal RC rhythm in CD8αβ^+^ LPLs, but did reduce the proportion of this cell population (Fig. 8a). CD4^+^ LPLs in GF and GF-mice following FMT remained unchanged, as also seen in RC- and WD-mice with and without microbial depletion (Fig. 8b vs Fig. 7b). Interestingly, the proportion of LPL B-cells was increased at the ZT14 time point in both GF and GF-FMT animals (Fig. 8c), despite no time-dependent patterns in RC-mice (Fig. 7c). In contrast, minimal changes were observed in the proportions of LPL myeloid cells in these animals (Fig. 8d).

## Discussion

The SI is the major site of dietary exposure, carrying out the critical roles of nutrient digestion and absorption^11^. Given the short lifespan of the IECs (3–5 days), the intestine has been deemed the most highly regenerative organ in the body, accounting for 20–30% of whole-body protein synthesis^38^. At the same time, the balance between IEL and LPL immune cell populations ensures that the SI can conduct immunosurveillance while maintaining an immunotolerant environment following dietary challenge. The findings of the present study demonstrate that both structure and immune functions of the SI exhibit patterns that are not only influenced by time-of-day, but also by diet and the presence of a normal intestinal microbiome.

Intestinal growth is affected not only by crypt cell proliferation, but also by IEC use of dietary nutrients as an energy source^38^. Hence, changes in intestinal growth, as determined by analyses of intestinal weight and crypt-villus height, have been observed in response to changes in nutrient intake in murine models of fasting and re-feeding^39,40^. Indeed, crypt-villus height is reduced by a remarkable 19% in 24-h fasted mice, and refeeding for 24-h completely restores the SI architecture^38^. Furthermore, an approximately 18% increase in small intestinal villus height within a 6-h period has been reported in piglets following feeding of medium chain triacylglycerol^41^. These studies are consistent with the 12% difference in villus height observed during the 12-h between the onset of fasting and onset of feeding observed herein. Similarly, following an extreme fasting period, food intake in snakes is associated with a doubling in intestinal mass as a result of a six-fold increase in microvillus length^42^. The concomitant increase in intestinal weight and villus height observed in RC-animals at ZT14, the onset of the feeding period in rodents, is therefore consistent with SI growth being induced by the increase in food intake.

The IEC that line the villi constitute the absorptive surface of the small intestine, and small intestinal surface area is thus the critical physiological determinant of the absorption of orally ingested substances, in rodents as well as humans^43–45^. For example, in the rat jejunum, the surface area of the villi amplifies that of the mucosa by 5-fold^43,44^. It can therefore be estimated that the 12% increase in villus height, as observed between ZT2 and ZT14, represents a 12% increase in absorptive capacity, even without taking into account any additional contributions by the microvilli, which amplify the absorptive surface area by an additional 21-fold^43,44^. Such analyses have largely been conducted to determine the translatability of studies on oral drug absorption from rodents to humans^44,45^ but, indeed, villus height positively correlates with nutrient absorption in a variety of different animals models^46,47^ as well as following administration of long-acting agonists of the intestinal growth factor, glucagon-like peptide-2 (GLP-2) in rodents and humans^48–50^. Furthermore, reductions in villus height, such as observed in congenital-, environmental- and infection-induced enteropathies are associated with malabsorption and diarrhea, some of which can be reversed by restoration of villus height^51–54^. Finally, a recent study has demonstrated using both gain- and loss-of-function approaches that a 25% increase in villus length and the associated expansion of intestinal surface area leads to enhanced absorption of dietary fat^55^. Therefore, in keeping with the evolutionary presentation of the circadian system, it can be hypothesized that the 12% increase in villus height observed herein during the transition from ZT2 to ZT14 is anticipatory, to enhance the absorptive capacity of SI and thus, ensure increased energy availability during the normal rodent feeding period.

Of importance, the increased intestinal weight, villus height, and crypt depth in RC-mice at ZT14 corresponds to the previously identified peak in circadian secretion of intestinal incretin hormone Glucagon-like Peptide-1 (GLP-1)^33^. GLP-1 receptor agonists are known to enhance SI weight through the induction of crypt fission^56^. However, co-secreted alongside GLP-1 from the enteroendocrine L-cell is the intestinotrophic hormone, GLP-2, which has been shown to stimulate crypt cell proliferation and inhibit IEC apoptosis, resulting in increased SI villus height and weight^49,57^. Furthermore, the disrupted rhythms in intestinal weight and villus height observed in WD-fed mice, coincide not only with a disrupted food intake pattern in these animals, but also with parallel changes in L-cell hormone secretion^33^. The larger SI observed in both GF- and RC-mice following antibiotic administration not only parallels the previously reported dramatic increase in basal L-cell secretion found in these animals^33^, but also regulation of intestinal hormone secretion. The microbiome has also previously been reported to impact growth related endocrine pathways such as the somatotrophic axis, altering the production and activity of insulin-like growth factor-1^58^. Hence, circadian- and diet-induced changes in intestinal L-cell secretion of GLP-1 and/or GLP-2 may contribute to the rhythmic changes in SI growth observed in normal mice, as well as in animals fed a high-fat diet and GF-mice.

SI mucosal expression of the core clock genes *Arntl* and *Per2* in RC-mice mimicked that of other known metabolic tissues, with a higher expression of *Arntl* at ZT2 as compared to ZT14 that was anti-phasic to that of *Per2*^34^*.* While obesogenic feeding has been shown to alter metabolic tissue clock gene expression^33,35,36^, the SI rhythm in *Arntl* was largely unaffected by either WD-feeding or disruption/absence of the microbiome as in the AIMD- and GF-mice, respectively. Therefore, clock gene expression in the heterogenous cell population that constitutes the intestinal mucosa appears to be regulated differentially as compared to metabolic tissues. It is therefore also possible that the observed changes in SI growth are independent of the IEC clock. However, a limitation of this conclusion is that changes in mucosal clock gene expression may not reflect those in the proliferating crypt base stem cells, which are also known to exhibit circadian rhythms^59^.

Immune function has been well established to be circadian in nature, as immune cell trafficking and cytokine levels differ over the course of the day^11,29^. Circulating levels of proinflammatory cytokines have been demonstrated to peak during the active phase in mice, whereas circulating leukocytes are highest during the rest phase and are recruited to tissues at the onset of the active phase^29,60^. As previously demonstrated in the intestine^27^, assessment of select cytokine genes in SI revealed elevated *Tnf* at the onset of the fasting/rest period in normal mice. While IECs have been shown to be influenced by changes in intestinal cytokine levels, no clear patterns were observed in the selected cytokine profile in relation to gravimetric and morphometric SI parameters^27,61^. Specifically, *Tnf* was found to change in a time-, diet-, and microbiome-dependent manner; however, expression did not parallel SI changes despite the fact that epithelial-derived *Tnf* has been speculated to boost Wnt signalling to promote IEC proliferation^62^. Furthermore, while obesogenic diets have been associated with an increased proinflammatory phenotype^63^, *Tnf* expression was reduced at ZT2 in WD-fed animals, disrupting the rhythm found in RC-fed mice. Short-chain fatty acids (SCFA) have been also been reported to modify cytokine production by IECs and, interestingly, previous analyses in RC-fed mice revealed a reduction in total cecal SCFA at ZT14, as well as decreases at both ZT2 and ZT14 following WD-feeding, providing a potential mechanism for altered *Tnf* expression^64^*.* This is further supported by the reduction in *Tnf* expression in AIMD-mice, which have an impaired ability to generate microbial metabolites. Furthermore, GF-mice lacked the rhythms in cytokine levels seen in normal mice, although FMT failed to restore these rhythms, a finding that requires further investigation.

Circadian rhythms observed within the immune system include those of leukocyte trafficking to peripheral tissues, which peak during the active/feeding period, likely to maintain adequate immunosurveillance during the period of maximal bacterial exposure^29,60^. Given that the active period is also associated with increased exposure to nutrient-derived luminal antigens, inappropriate immune responses are thought to be inhibited by the upregulation of CD8αα on intestinal lymphocytes^28,65^. Previous reports have demonstrated diurnal changes in SI IELs, wherein the ratio of CD8αα homodimer- to CD8αβ heterodimer-expressing cells is greater in the rodent active phase^66^. In keeping with these findings, the increase in CD8αα^+^ cells in association with the decrease in proportion of CD8αβ^+^ cells at the onset of the feeding period in RC-mice reported here, may play a physiological role in maintaining immunological tolerance in the SI. Furthermore, the disrupted pattern in WD-IELs, is likely a response to their disruption in food intake pattern^33^. Interestingly, recent studies have identified the GLP-1 receptor on IELs and have demonstrated their role regulating GLP-1 bioavailability^67^. Consistently, parallel changes were observed in the proportion of CD8αβ^+^ cells and the previously-reported stimulation of GLP-1 secretion in both RC- and WD-mice with or without AIMD and in GF-mice with or without FMT^33^. Finally, CD8αβ^+^ LPLs displayed an opposing pattern to the IELs, likely indicative of lymphocyte homing from the lamina propria to the epithelium^28^.

Given the unique interaction between the microbiome and the intestinal immune system, as well as the differential microbial composition and function along the length of the GI tract^68^, analysis of the SI microbiome would be warranted to infer specific microbial-driven changes in intestinal immunity. Furthermore, while GF-mice were employed to study the rhythmic nature of select parameters of intestinal homeostasis in the absence of a microbiome, in general, FMT failed to re-establish patterns observed in normal RC-mice. A limitation of the present study is the lack of FMT experiments using WD-fed mice microbiome, as well as the use of pooled RC fecal samples, which precludes interrogation of the effects of the normal day vs. night pattern in microbial composition; both of these issues warrant future consideration. Furthermore, the overall inability of the RC FMT to restore the physiologic rhythms in the observed intestinal parameters may be a result of the variation in the relative abundance of major families between normal RC-mice and the recolonized GF-animals. The use of aerobic- rather than anaerobically-reduced PBS to resuspend the RC feces may have contributed to this variation through reductions in strict anaerobes. However, while an important tool for microbiome research, GF-animals have been demonstrated to have impairments in both early development and education of the intestinal immune compartment^32^. As such, conducting FMT studies using AIMD adult mice might further support of the role of the microbiome in maintaining SI responses over the 24-h day and in response to WD-feeding.

In addition, time-restricted feeding, as well as the implementation of reverse light/dark cycle studies would also provide further insight into the relationship between feeding patterns and intestinal homeostasis. Notwithstanding, the findings of this study enhance our current understanding of diurnal SI changes in both structure and immune cell populations, showing that these are driven by dietary composition and timing, as well as by the presence of a normal microbiome. Such insights may explain the increased risk for intestinal dysfunction in individuals suffering from circadian disruption or obesity.

## Methods

### Animal models

Male and female C57Bl6/J mice (5–7 weeks) were purchased from Jackson Laboratories, and randomized into treatment groups upon receipt and allowed to acclimate to the University of Toronto animal facility for one week prior to the beginning of the experimental conditions. For the duration of the experiment, mice were housed in a 12/12-h light/dark cycle at constant room temperature with free access to water, and were fed either regular chow (RC (n = 24 with 12 mice used per time point); 18%, 58%, and 24% calories from fat, carbohydrate and protein, respectively; overall caloric density of 3.1 kcal/g [2018 Envigo]) or a high-fat/high-sucrose Western diet (WD (n = 24 with 12 mice used per time point); 41%, 43%, and 17% calories from fat, carbohydrate [29% sucrose, 14% other], and protein, respectively; overall caloric density of 4.7 kcal/g [D120798, Research Diets]) for 16 weeks to establish a model of diet-induced obesity. To achieve antibiotic-induced microbial depletion (AIMD), mice (n = 12 with 6 mice used per time point) were orally gavaged twice-daily with either vehicle (water) or an antibiotic/antifungal cocktail (ampicillin (100 mg/kg), vancomycin (50 mg/kg), metronidazole (100 mg/kg), neomycin (100 mg/kg), amphotericin B (1 mg/kg)) during the final 2 weeks of feeding^33,69^. Male (n = 7–8 with 3–4 mice used per time point) and female (n = 7–8 with 3–4 mice used per time point) RC germ-free mice on a C57Bl/6 N background were bred under standard germ-free conditions prior to transfer to a sterile 12/12-h light/dark cycle for fecal microbiome transfer (FMT). In brief, fecal pellets from RC-animals were collected over 24-h and resuspended in normal, oxygenated PBS (200 μL; 5 g/mL) prior to oral gavage daily for 2 days into 8-wk old germ-free mice, followed by a 3-week rest period. The sample size for all experiments was determined based on previous studies of the same nature conducted in our laboratory^33^. All animal studies were approved by the University of Toronto Animal Care Committee and conformed to the Canadian Council on Animal Care guidelines. All of the protocols, as well as the results of the fecal 16S rRNA gene sequencing, cecal bile acid and short chain fatty acid, and metabolic data in these mice have been previously reported^33^.

### Gravimetric and morphometric analysis

The small intestine was isolated, rinsed with ice-cold PBS, and both the weight and length (under constant tension using a 2.8 g weight) were measured. A two cm section from the ileum was collected into formalin for paraffin-embedding, sectioning and staining with hematoxylin & eosin (Pathology Research Program, University Health Network, Toronto, ON). Ileal villus height and crypt depth were measured in blinded fashion using a Zeiss microscope with AxioVision software (Zeiss Microscopy Canada), with at least 20 well-oriented crypts and villi measured from each animal, to make n = 1.

### Gene expression analysis

RNA was isolated from ileal mucosal scrapes using the RNeasy Plus Mini Kit (Qiagen Inc.) and cDNA was generated using 5 × All-in-One RT Mastermix (Applied Biological Materials Inc.). Quantitative reverse-transcriptase polymerase chain reaction (qRT-PCR) was performed using the Taqman Gene Expression Assay (ThermoFisher) with primers (ThermoFisher) as listed in Table 1.

**Table 1:** List of primer sequences.

Target	Product number
*Arntl*	Mm00500226_m1
*Per2*	Mm00478113_m1
*Tnf*	Mm00443258_m1
*Ifng*	Mm01168134_m1
*Il6*	Mm00446190_m1
*Il10*	Mm01288386_m1
*Tgfb*	Mm01178820_m1
*18S*	Hs99999901_s1

### Lymphocyte collection and flow cytometry

In brief, IELs and LPLs were isolated by dividing the distal 10 cm of the small intestine into two cm segments and incubating in HBSS with 5 mM DTT to remove mucus. Two consecutive 10 min incubations with 5 mM EDTA were performed to collect the intestinal epithelial cells and associated IELs after debris removal using a tube-top 40 µm strainer. To collect the LPLs, 3 additional EDTA incubations were conducted followed by mincing of the tissue, enzymatic digestion with 0.2 U/ml Liberase TM (Roche) and 200 U/ml DNAse I (Roche) for 30 min, trituration of the digested tissue through a 21G needle and filtration to remove debris^70^.

Single cell suspensions were stained with LIVE/DEAD fixable aqua dead cell stain (ThermoFisher) to exclude dead cells from flow cytometric analysis. Suspensions were then incubated in flow cytometry staining buffer (0.3%FBS, 0.1% NaAzide) with Anti-Mo CD16/CD32 (ThermoFisher, Clone 93) to block non-specific binding of antibodies by Fc receptors. Antibodies (ThermoFisher) listed in Table 2 were used for staining of cell surface antigens, following which, the eBioscience Foxp3/Transcription Factor Staining Kit (ThermoFisher) was used for fixation. Analysis was conducted using the LSRFortessa Cell Analyzer (BD Biosciences) running FACSDiva acquisition software, and data were analyzed using FlowJo v10^71^.

**Table 2:** List of antibodies.

Target	Fluorophore
CD45	PerCP-Cy5.5
CD3	PE-Cy7
TCRb	eFluor 450
CD8α	Alexa Fluor 700
CD8β	APC
CD4	APC-eFluor 780
CD19	PE-eFluor 610
CD11b	Super Bright 780
Ly6g	FITC

### Statistical analysis

Statistical analysis was conducted using GraphPad Prism. Data were analyzed for significance by 2-way ANOVA followed by appropriate post hoc analysis for 3 or more groups, or by Student’s t-test for two groups. Statistical outliers were determined using the Grubbs’ test. All data are expressed as mean ± SEM.

## Supplementary Information

Supplementary Information.

## Data Availability

The datasets generated during and/or analysed during the current study are available from the corresponding author on reasonable request.

## Abbreviations

AIMD: Antibiotic-induced microbial disruption
GF: Germ-free
GI: Gastrointestinal
GLP-1: Glucagon-like peptide-1
IEC: Intestinal epithelial cell
IEL: Intraepithelial lymphocytes
LPL: Lamina propria lymphocytes
RC: Regular chow
SI: Small intestine
WD: Western diet
