# Supplementary material for: Diurnal changes in the murine small intestine are disrupted by obesogenic Western Diet feeding and microbial dysbiosis
Source: Sci Rep. 2021 Oct 18;11:20571. doi: 10.1038/s41598-021-98986-7 (PMC8523685; doi:10.1038/s41598-021-98986-7)

Diurnal changes in the murine small intestine are disrupted by obesogenic Western Diet feeding and microbial dysbiosis

Sarah E. Martchenko[1], David Prescott[2,3], Alexandre Martchenko[1], Maegan E. Sweeney[1], Dana J. Philpott[2], Patricia L. Brubaker[1,4]

Departments of [1]Physiology, [2]Immunology, [3]Laboratory Medicine and Pathobiology, and [4]Medicine, University of Toronto,

Toronto, ON Canada

Supplementary Figure 1: Representative 10X images of intestinal cross-sections from RC- and WD-fed animals with and without AIMD, at ZT2 and ZT14.

Supplementary Figure 2: Relative abundance of the top 10 families in colonic feces from RC- and WD-fed mice with and without AIMD, at ZT2 and ZT14. n=6 male and n=6 female mice.

Supplementary Figure 3: Relative abundance of the top 10 families in colonic feces from GF-mice following FMT with RC-microbiome, at ZT2 and ZT14. n=3-4 male and n=3-4 female mice.

Supplementary Figure 4: Representative 10X images of intestinal cross-sections from GF and GF-mice following FMT with RC-microbiome (GF+RC), at ZT2 and ZT14.

Supplemental Figure 1

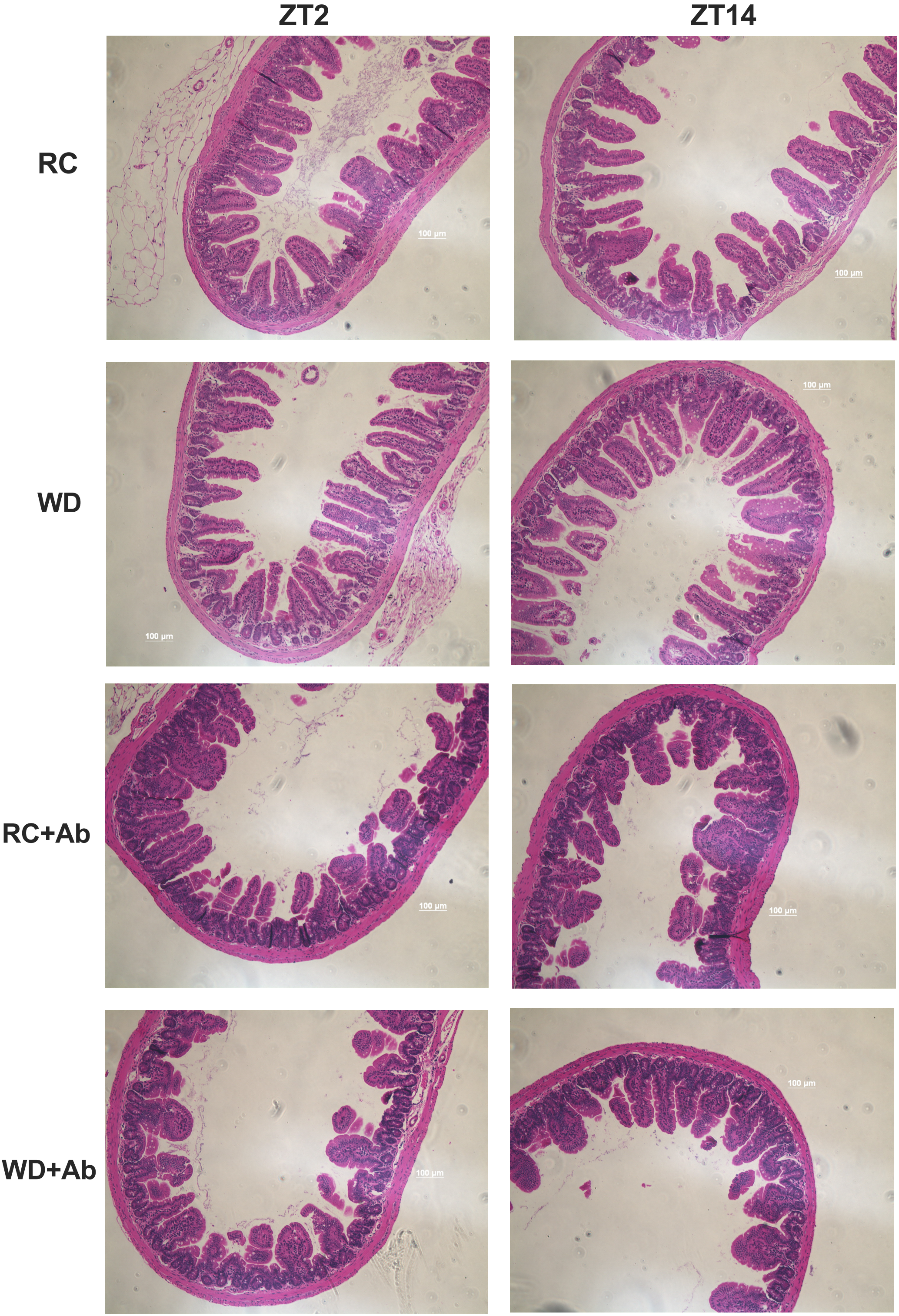

Supplemental Figure 2

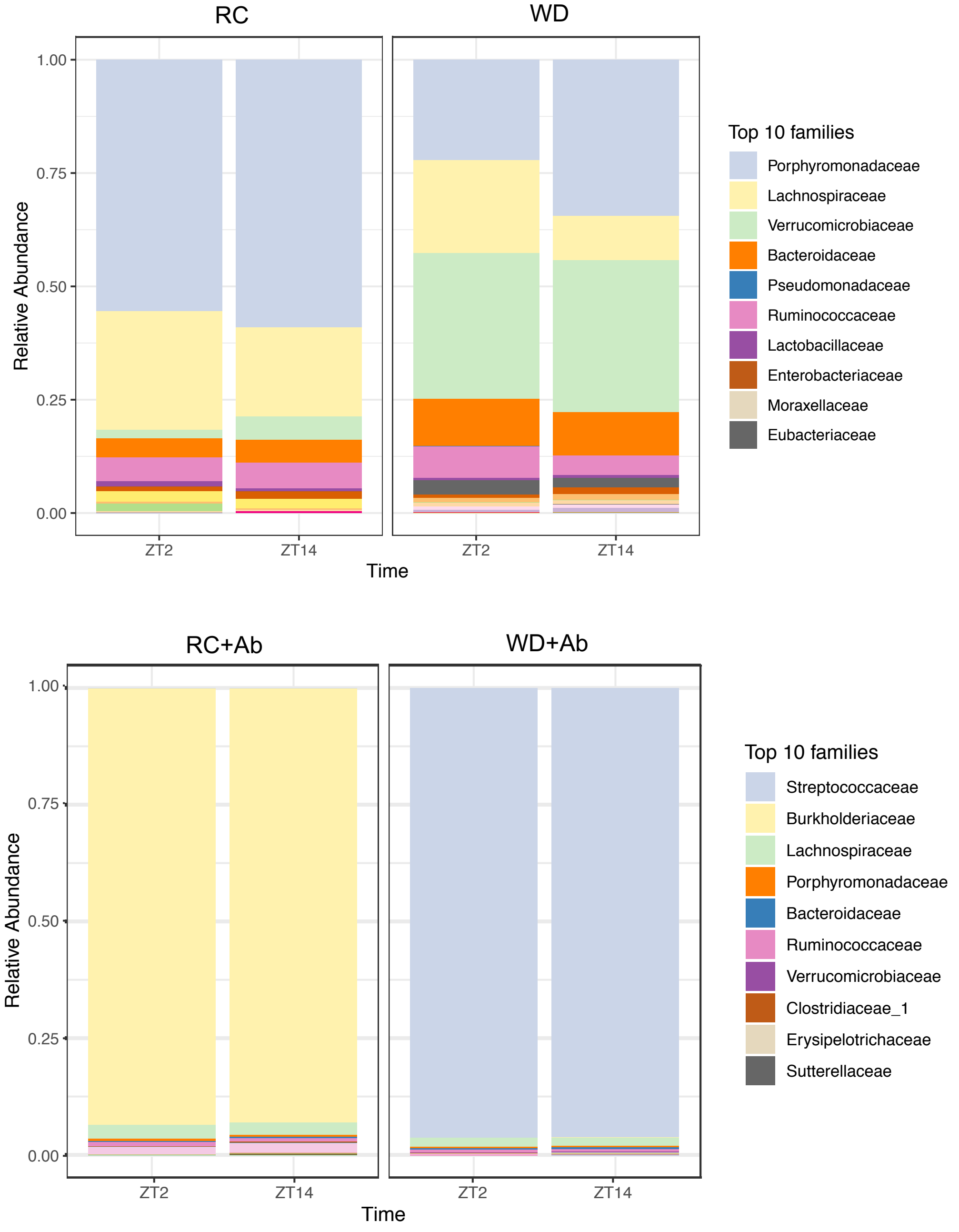

Supplemental Figure 3

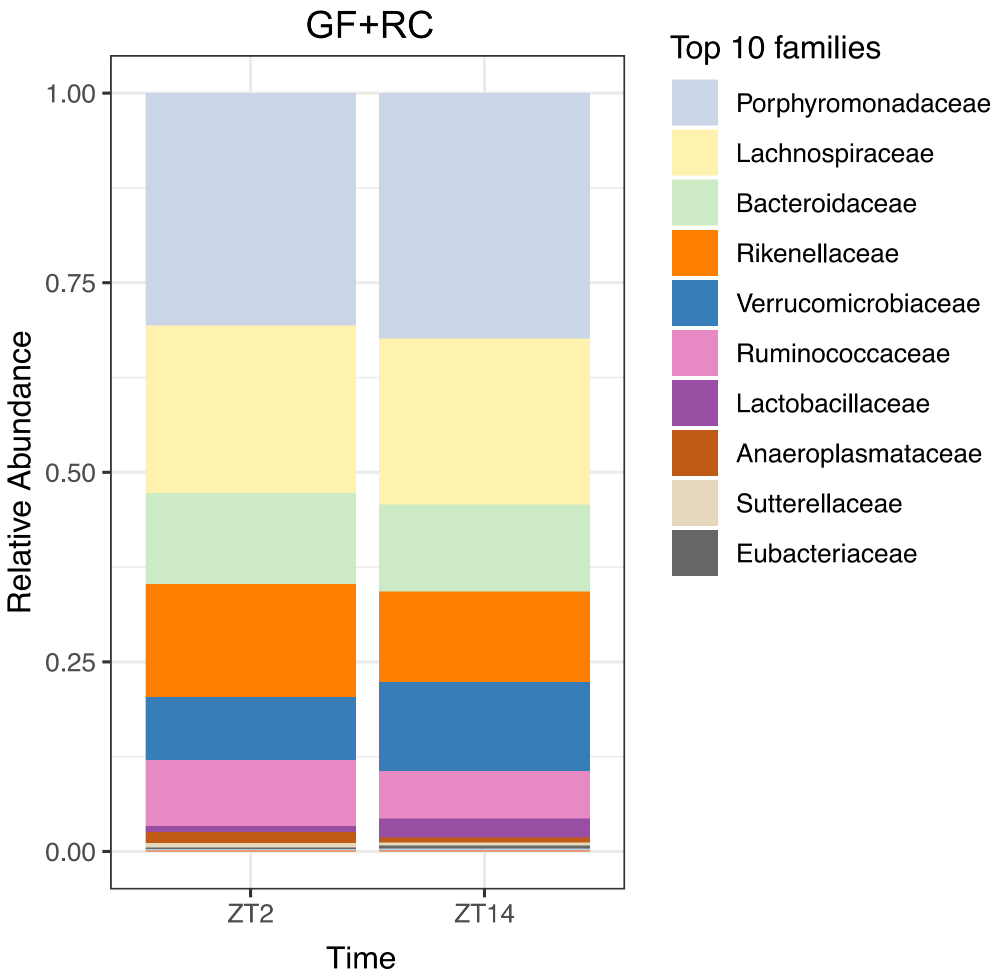

Supplemental Figure 4

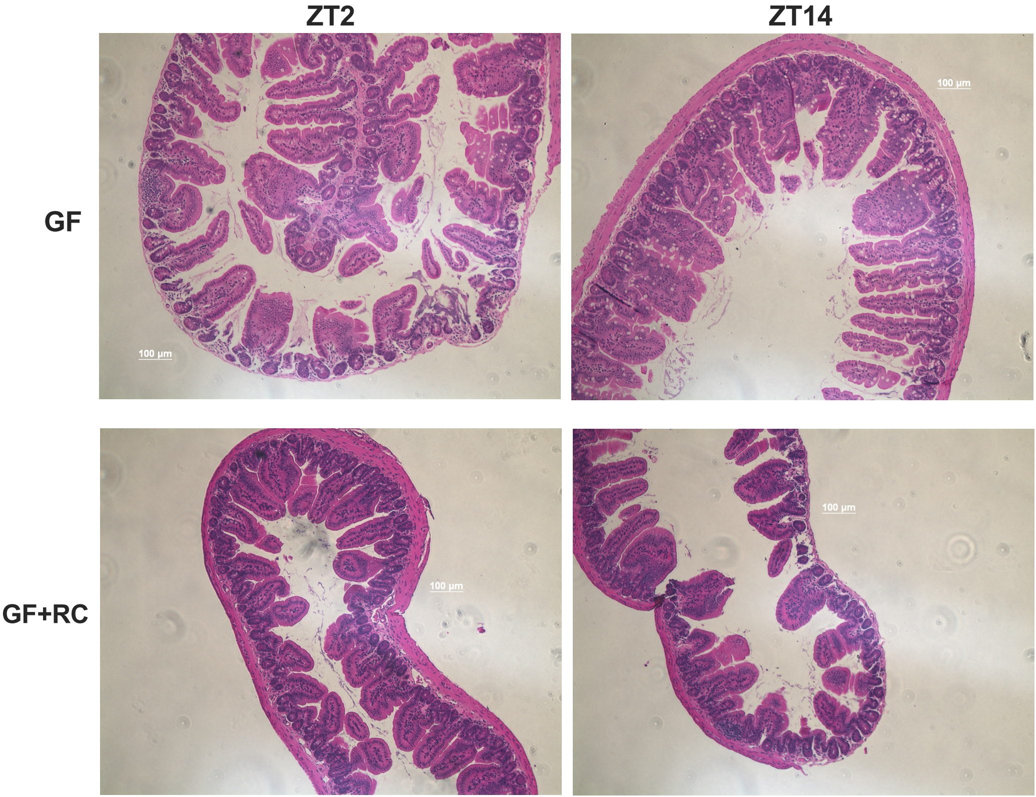

Supplement: Supplementary file 1 — Supplementary Information. [file 41598_2021_98986_MOESM1_ESM.pdf]
